# Genetic diversity and population structure of the rockpool shrimp *Palaemon elegans* based on microsatellites: evidence for a cryptic species and differentiation across the Atlantic–Mediterranean transition

**DOI:** 10.1038/s41598-020-67824-7

**Published:** 2020-07-01

**Authors:** Inés González-Castellano, Jorge González-López, Ana M. González-Tizón, Andrés Martínez-Lage

**Affiliations:** Departamento de Biología and Centro de Investigaciones Científicas Avanzadas (CICA), Universidade da Coruña, 15071 A Coruña, Spain

**Keywords:** Genetic markers, Population genetics, Biogeography, Molecular ecology, Evolutionary genetics

## Abstract

The rockpool shrimp *Palaemon elegans* is an ecologically important crustacean species within the European coastline fauna. In the present study, genetic diversity and population structure and connectivity were assessed by examining 21 polymorphic microsatellite loci at 13 sampling sites located along the natural distribution range. All localities showed similar levels of genetic variability. Significant deficits of heterozygosity were recorded, most likely due to the presence of null alleles. Genetic structure analyses revealed two clearly genetically distinct groups within *P. elegans* but without following any geographical or oceanographic basis. Thus, our results provided nuclear evidence for the existence of a Mediterranean cryptic species within *P. elegans,* highlighting the need to revise its taxonomic status. Regarding *P. elegans sensu stricto*, population structuring was reported across the Atlantic–Mediterranean transition area, where the Almería-Orán Front restricts the gene flow between the Atlantic and the Mediterranean population. Moreover, while population connectivity was suggested between all Mediterranean localities, some substructure was found within the Atlantic group. Canary Islands exhibited a weak but significant genetic differentiation from all Atlantic mainland localities, consistent with the isolation-by-distance pattern detected throughout the Atlantic population. Overall, all these findings provided new insights into the population biology of *P. elegans* complex.

## Introduction

The rockpool shrimp *Palaemon elegans* Rathke, 1837 is a crustacean decapod common in tidal rockpools, *Zostera*, *Posidonia* and *Cymodocea* sea grasses meadows and it also can be found in hypersaline lagoons and in slightly brackish water close to river mouths^1^. This species is characterized by its capability to adapt to highly variable environments, as it can cope with a wide range of salinities, temperatures and oxygen conditions^2,3^. Regarding to the native geographical distribution of *P. elegans*, it ranges from the eastern Atlantic Ocean (from Scotland and Norway to Mauritania, including the Azores, Madeira and Canary Islands) to the entire Mediterranean Sea and the Black Sea^4^. Unintentional introductions resulted in this shrimp inhabiting the Caspian Sea and the Aral Sea nowadays. Similarly, *P. elegans* was introduced in the Baltic Sea, replacing the native counterpart *P. adspersus* in that region^5,6^. *Palaemon elegans* has been also recently reported to occur on the northeast coast of the United States^7^, so is currently distributed beyond European waters. Thus, even though *P. elegans* is considered an important species within the European coastline fauna due to its broad ecological niche and ongoing geographic expansion^8^, population genetics analyses are scarce.

Genetic breaks occur when there is a marked genetic differentiation in a continuously distributed species^9^. For many years, high levels of gene flow associated with little genetic structuring were assumed for marine species with planktonic larval phases^10^ as *P. elegans*. However, it has been proved that historical and oceanographic processes could drive genetic differentiation between marine populations, even in species with high dispersal abilities^11^. This is the case of the shrimp *P. elegans* since Reuschel et al*.*^8^ carried out a phylogeographic analysis using two mitochondrial DNA (mtDNA) markers, revealing the existence of three haplogroups, one of them in the Atlantic localities (type I) and two from the Mediterranean localities (types II and III). In that study, a genetic break was found in the Atlantic–Mediterranean transition area, restricting the gene flow between these basins and leading to genetic differentiation between the Atlantic (type I) and the Mediterranean (type II) population. Similar phylogeographic breaks across the Atlantic–Mediterranean transition have been reported in a wide variety of species (see review in Patarnello et al*.*^11^). These genetic discontinuities are frequently attributed to the effect of different oceanographic discontinuities acting in this area, i.e. the Almería-Orán Front, the Strait of Gibraltar, the Balearic Front or the Ibiza Channel^10^.

Back to *P. elegans*, genetic structure was not detected by Reuschel et al*.*^8^ neither within the Atlantic population (type I) nor within the Mediterranean one (type II). As for *P. elegans* type III, it was only recorded from Mediterranean localities and showed high nucleotide divergence from the other two types^8^. This genetic pattern was confirmed in later phylogeographic studies in the Mediterranean Sea also using mtDNA markers^12,13^. Reuschel et al*.*^8^ proposed that type III individuals could belong to a putative cryptic species existing within *P. elegans*. Cryptic species occur when two or more species are classified as a single nominal species because they are superficially morphologically indistinguishable^14^. The Mediterranean Sea is characterized by a high level of biodiversity and species richness with a proportion of endemism circa 25%^15^, largely related to its tortuous geological history. Consequently, cryptic speciation is not considered an uncommon phenomenon in the Mediterranean Sea and molecular markers have indicated the presence of cryptic species in several taxa including shrimps^16,17^. Given these findings in *P. elegans,* the need for further genetic studies using polymorphic nuclear markers, such as microsatellites, to clarify its phylogeography was highlighted.

Microsatellite markers have been used in a wide range of applications in population genetics, ecological, conservation and evolutionary studies^18^ because of its codominant nature, biparental mode of inheritance and high levels of polymorphism^19^. Specifically in population genetics, microsatellites are extremely valuable tools as they might reveal the existence of genetically distinct populations even in fine-scale studies^20^. In fact, microsatellite loci have been crucial in understanding the population structure of several shrimp species as *Penaeus vannamei*^21^, *Penaeus monodon*^22^, *Pandalus borealis*^23^, *Aristeus antennatus*^24^ or *Palaemon serratus*^25^. Thanks to the advent of high-throughput sequencing technologies, the development of microsatellite markers in non-model species has become a rapid and cost-effective process^26^. Precisely, we isolated and characterized 21 novel polymorphic microsatellite loci for *Palaemon elegans* using Illumina MiSeq sequencing^27^.

The aim of the present study was to address for the first time the genetic diversity and population structure and connectivity of the shrimp *P. elegans* along the European coastlines, with particular attention to the Atlantic–Mediterranean transition, using microsatellite loci previously developed^27^ to shed light about the population biology of this species.

## Results

### Genetic diversity

A total of 400 individuals were genotyped for 21 microsatellite markers isolated and characterized in a previous work^27^. Thirteen coastal localities distributed along the natural distribution range of *P. elegans* were sampled. The number of individuals collected from each sampling site varied from 10 (Ebro Delta) to 50 (Collioure). No evidence of linkage disequilibrium between pairs of loci was detected across localities after the sequential Bonferroni correction. Hence, all microsatellites were considered as independent markers. Likewise, the Ewens–Watterson’s neutrality test confirmed that all loci were selectively neutral. Estimators of genetic variability per locality, per locus and per locality and locus are available in Table 1, Supplementary Tables S1 and S2, respectively. Across the microsatellite loci, the number of alleles ranged from 4 (loci Pe04, Pe13 and Pe20) to 25 (locus Pe06), with a grand mean of 5.3 alleles over localities and loci. Thirty-six out of the 217 identified alleles (16.59%) were private alleles. Every microsatellite locus showed private alleles from at least one sampling site (except loci Pe02, Pe03, Pe08 and Pe13 with no private alleles), and every locality showed private alleles in at least one locus (except Lanzarote) (Table 1 and Supplementary Table S1). Average allelic richness (*AR*) across localities ranged from 2.032 (Santoña) to 2.453 (Livorno). The 21 microsatellites were highly polymorphic as the mean percentage of polymorphism was 98.53% (Table 1). The observed heterozygosity (*Ho*) over localities for each locus ranged from 0.134 (Pe09) to 0.842 (Pe14), and the expected heterozygosity (*He*) from 0.201 (Pe09) to 0.876 (Pe14) (Supplementary Table S1). Regarding the expected and observed heterozygosity for each sampling site, *Ho* ranged from 0.372 (Santoña) to 0.499 (Ebro Delta) and *He* from 0.468 (Santoña) to 0.607 (Livorno). *Ho* and *He* averaged 0.450 and 0.531, respectively (Table 1).

**Table 1:** Genetic diversity for 13 sampling sites of *Palaemon elegans.*

	Locality	*N*	*Na*	*A*	*AR*	*P* (%)	*Ho*	*He*	*FIS*
Atlantic Ocean	Ré Island	30	96 (1)	4.571	2.081	95.24	0.448	0.492	0.112
	Santoña	30	95 (2)	4.524	2.032	100.00	0.372	0.468	0.221
	Ártabro Gulf	45	110 (4)	5.238	2.071	100.00	0.448	0.491	0.100
	Cádiz	40	112 (5)	5.333	2.101	100.00	0.439	0.504	0.141
	Tenerife	38	111 (1)	5.286	2.178	100.00	0.481	0.529	0.104
	Lanzarote	30	103 (0)	4.905	2.186	95.24	0.483	0.528	0.103
Mediterranean Sea	Granada	40	129 (2)	6.143	2.348	100.00	0.474	0.591	0.207
	Almería	29	114 (2)	5.429	2.255	100.00	0.434	0.548	0.199
	Ebro Delta	10	95 (1)	4.524	2.298	100.00	0.499	0.539	0.127
	Collioure	50	137 (5)	6.524	2.317	100.00	0.494	0.571	0.143
	Marseille	18	106 (3)	5.048	2.219	100.00	0.422	0.518	0.212
	Livorno	20	132 (4)	6.286	2.453	100.00	0.446	0.607	0.284
	Mallorca	20	110 (6)	5.238	2.242	90.48	0.418	0.521	0.226
	Total/average	400	217 (36)	5.311	2.214	98.53	0.450	0.531	0.168

*N*, number of sampled individuals; *Na*, number of alleles per locality, with private alleles in brackets; *A*, mean number of alleles per locality; *AR*, allelic richness; *P*, percentage of polymorphism; *Ho*, mean observed heterozygosity; *He*, mean expected heterozygosity; *FIS*, inbreeding coefficient.

After the sequential Bonferroni correction, 242 out of the 273 locality-locus combinations (88.64%) showed no significant deviation from Hardy–Weinberg equilibrium (HWE). Nine out of the 21 loci (Pe03, Pe04, Pe05, Pe07, Pe12, Pe13, Pe15, Pe20 and Pe21) conformed to HWE in all localities, meanwhile the remaining 12 loci were out of HWE in at least one locality (Supplementary Table S2). Regarding localities, Collioure was the locality where more loci deviated from HWE, while in Lanzarote, Ebro Delta and Marseille no loci were significantly out of HWE. Most of these combinations departing from HWE were accompanied by positive *FIS* values, indicating the existence of a heterozygote deficit. An excess of heterozygotes from Collioure for locus Pe02 was detected and from Livorno for locus Pe10. Null allele frequencies were mostly low, varying from 0.022 to 0.226 across loci. Only one locus, the locus Pe11, exhibited a high null allele frequency, > 0.2 according to Chapuis & Estoup^28^, over localities (Supplementary Table S1). In detail, eight loci (Pe01, Pe02, Pe06, Pe08, Pe11, Pe17, Pe18 and Pe19) showed high null allele frequency (above 0.2) in at least one locality (Supplementary Table S2), being again the locus Pe11 the one with null allele frequency > 0.2 in more localities (Ré Island, Santoña, Ártabro Gulf, Tenerife, Collioure, Marseille and Livorno). Given the consistency in the presence of null alleles across localities, locus Pe11 was not included in the further population structure analyses. Specifically, 14 out of the 31 locality-locus combinations departing from HWE (48.28%) showed evidence of presence of null alleles in a frequency higher than 0.2, whilst in the remaining combinations the proportion of null alleles was lower (Supplementary Table S2).

### Genetic structure

**Figure 1:**
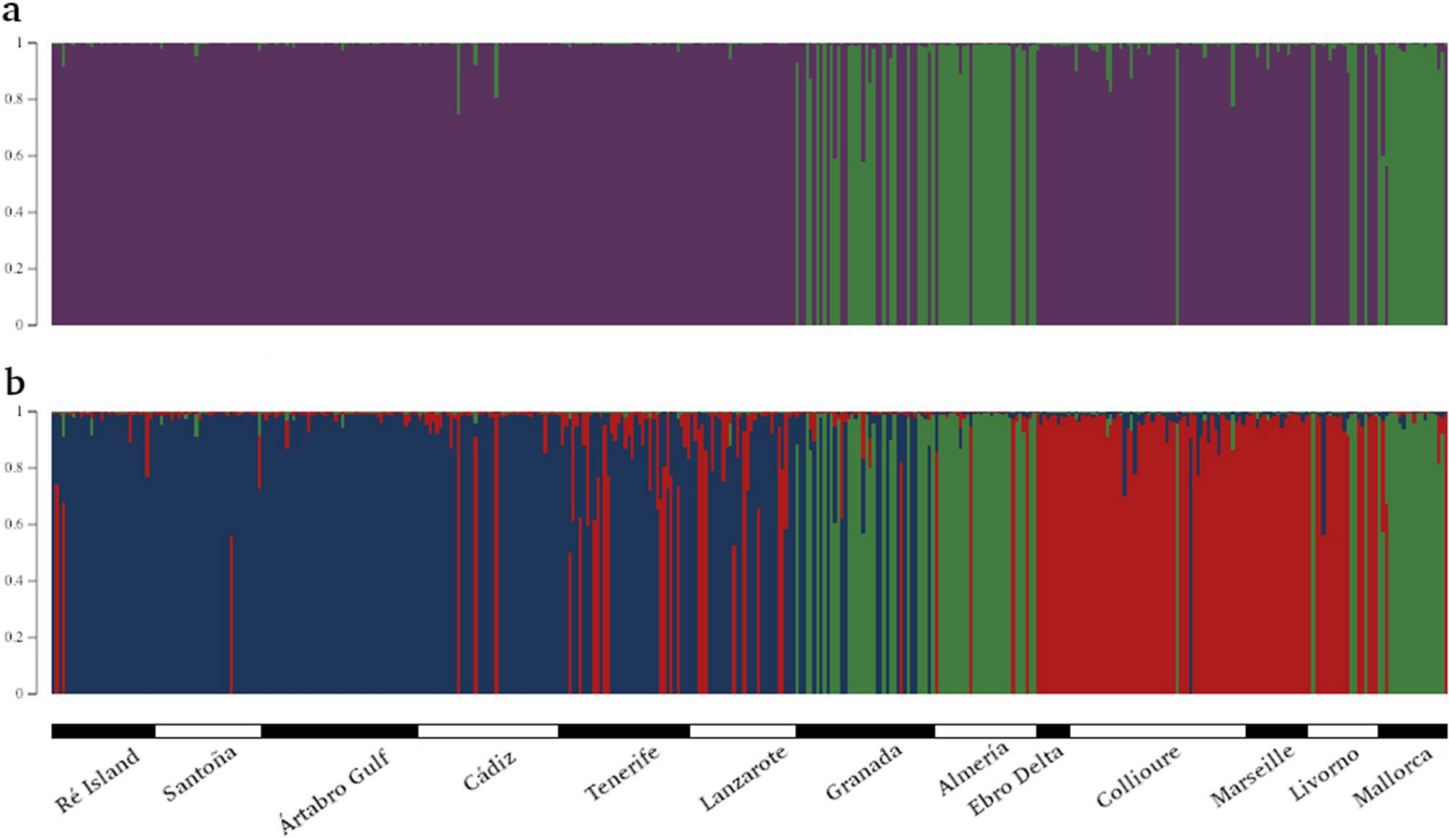
Bayesian assignment probabilities for (**a**) *K* = 2 and (**b**) *K* = 3 revealed by STRUCTURE based on 20 microsatellite loci. Analysis including the 400 sampled individuals. Each color depicts a cluster and each vertical bar represents an individual with the probability of membership to a cluster. Colors correspond to (**a**) *P. elegans sensu stricto*: purple, and cryptic species: green; and (**b**) *P. elegans sensu stricto* (blue and red), and cryptic species (green)”.

The assessment of the modal value of Delta*K* by the STRUCTURE analysis (Supplementary Figure S1a) indicated that the most likely partition of the microsatellite dataset (400 sampled individuals) was two genetically distinct groups (*K* = 2, Fig. 1a). Most of the individuals (327 individuals) were assigned to one cluster, whereas the second cluster grouped 69 individuals. Accordingly, only 4 out the 400 sampled individuals could not be assigned to one specific cluster (probability of membership to a cluster < 0.75), most likely because they presented missing data at several loci. This partition in two groups did not follow any geographical or oceanographic pattern because the second cluster was formed by only some of the individuals collected in Granada, Almería, Collioure, Livorno and Mallorca, being the remaining individuals from these localities located in the main cluster. Factorial correspondence analysis (FCA) also showed genetic structuring in two main groups (Supplementary Figure S2) that coincided and supported the clustering inferred by STRUCTURE. Taking into account that the smaller cluster was exclusively formed by Mediterranean individuals, coupled with the findings reported by Reuschel et al*.*^8^, we considered that this group is the putative cryptic species suggested to exist within *P. elegans* with mtDNA markers. Consequently, to check if there was such agreement between our nuclear markers and the previously analysed mtDNA markers with respect to the 69 individuals of the smaller cluster, we generated the cytochrome c oxidase subunit I (COI) sequence from them following the PCR conditions described in Reuschel et al*.*^8^. A COI fragment was successfully amplified for 64 out of the 69 individuals. BLASTn verified that the COI sequence of all these individuals corresponded to the COI sequence of those individuals named *P. elegans* type III (HE573177) and considered as a cryptic species in Reuschel et al*.*^8^. Therefore, the assignment of 69 individuals of our dataset to a putative cryptic species by the 20 microsatellite loci was also supported with a mtDNA marker. COI sequences from the individuals belonging to the putative cryptic species can be easily accessed in Supplementary File S1. In detail, distribution of the putative cryptic species across localities identified by the Bayesian analysis yielded: 22 out of the 40 individuals collected in Granada (55% sampled individuals), 25 out of the 29 individuals collected in Almería (86%), 1 out of the 50 individuals collected in Collioure (2%), 4 out of the 20 individuals collected in Livorno (20%), and 17 out of the 20 individuals collected in Mallorca (85%).

In accordance with the STRUCTURE analysis, a peak of *ΔK* and a plateau in the plot of *K* versus ln Pr(X|*K*) (Supplementary Figure S1b) were also observed at *K* = 3, supporting a second partition of the data in three groups (Fig. 1b). One of these groups matched with the cluster corresponding to the putative cryptic species defined in *K* = 2. However, in this analysis, the individuals considered as *P. elegans sensu stricto* were divided into two clusters: individuals from Atlantic localities (Ré Island, Santoña, Ártabro Gulf, Cádiz, Tenerife and Lanzarote) and Granada were assigned to one of them, and individuals from the Mediterranean Sea (Almería, Ebro Delta, Collioure, Marseille, Livorno and Mallorca) to the other.

When all sampling sites were analysed separately, a hierarchical locus-by-locus AMOVA (Table 2a) revealed that the proportion of global genetic variance was mostly attributed to differences among individuals within localities (83.60%), whereas the proportion of genetic variance among sampling sites was 16.40% (*FST* = 0.164). More AMOVA analyses were performed to test the grouping scenarios inferred by STRUCTURE. Granada, Almería, Collioure, Livorno and Mallorca localities, with individuals belonging to the putative cryptic species, were subdivided by samples of each species (i.e. *P. elegans sensu stricto* and cryptic species) to conduct these analyses. Two-group AMOVA (*P. elegans sensu stricto* vs. cryptic species; Table 2b) maximized the *FCT*, revealing that the 26.23% of the total variance was explained by differences between *P. elegans sensu stricto* and the putative cryptic species. A minor but considerable proportion of the global variance (20.06%) was explained by differences among groups in three-group AMOVA (Atlantic localities and Granada of *P. elegans sensu stricto* vs. Mediterranean localities of *P. elegans sensu stricto* vs. localities of cryptic species; Table 2c). Nevertheless, a genetic structuring of the whole genotype dataset in two groups yielded a higher *FCT* value.

**Table 2:** Analysis of molecular variance (AMOVA) based on different grouping hypotheses.

Grouping	Source of variation	Percentage of variation	*F*-statistics
(a) None	Among localities	16.40	
	Within localities	83.60	*FST* = 0.164
(b) *P. elegans s. str.* vs. cryptic species	Among groups	26.23	*FCT* = 0.262
	Among localities within groups	5.41	*FSC* = 0.073
	Within localities	68.36	*FST* = 0.316
(c) Atlantic *P. elegans s. str.* vs. Mediterranean *P. elegans s. str.* vs.cryptic species	Among groups	20.06	*FCT* = 0.201
	Among localities within groups	2.69	*FSC* = 0.034
	Within localities	77.25	*FST* = 0.227

Analyses including the 400 sampled individuals. (a) No grouping indicates that localities were analysed separately. (b) For two-group analysis, localities with *P. elegans sensu stricto* individuals (*P. elegans*) and localities with putative cryptic species individuals (cryptic species) each formed a group. (c) For three-group analysis, localities with *P. elegans sensu stricto* individuals were pooled as Ré Island-Santoña-Ártabro Gulf-Cádiz-Tenerife-Lanzarote-Granada (Atlantic *P. elegans s. str.*) and Almería-Ebro Delta-Collioure-Marseille-Livorno-Mallorca (Mediterranean *P. elegans s. str.*), meanwhile localities with putative cryptic species individuals formed a third group.

Pairwise *FST* values were estimated between localities in which at least 10 individuals were sampled. Therefore, separating samples of Granada, Almería, Collioure, Livorno and Mallorca by species, *FST* values could not be calculated for some of these subdivided localities. Even so, pairwise *FST* values (Supplementary Table S3) suggested a marked and significant genetic differentiation in all the comparisons between localities of *P. elegans sensu stricto* and localities of cryptic species (*FST* ≥ 0.190), indicating the genetic divergence of these two species supported by the other analyses. Focussing on the population structure of *P. elegans sensu stricto*, pairwise *FST* values revealed significant genetic differentiation between the Atlantic and the Mediterranean localities (Table 3). Granada locality was connected with almost all the Atlantic localities and significantly differentiated from the remaining Mediterranean ones. Within the Mediterranean Sea, connectivity among Ebro Delta-Collioure-Marseille-Livorno was suggested by pairwise *FST* values. Along the Atlantic coast, connectivity was suggested among Ré Island-Santoña-Ártabro Gulf-Cádiz-Granada and some genetic differentiation was supported between each one of those and the Canarian localities. In other words, Tenerife and Lanzarote showed significant differentiation with all the localities regardless of the sampling source, while they were both connected to each other.

**Table 3:** Pairwise *FST* values (below the diagonal) between 11 sampling sites of *Palaemon elegans sensu stricto* (327 sampled individuals) as revealed by the microsatellite dataset (20 loci), and the corresponding *P*- values (above the diagonal).

		Atlantic Ocean						Mediterranean Sea				
	Locality	Ré Island	Santoña	Ártabro Gulf	Cádiz	Tenerife	Lanzarote	Granada	Ebro Delta	Collioure	Marseille	Livorno
Atlantic Ocean	Ré Island	–	0.001*	0.175	0.210	0.000**	0.000**	0.096	0.000**	0.000**	0.000**	0.000**
	Santoña	0.022	–	0.135	0.029	0.000**	0.000**	0.550	0.000**	0.000**	0.000**	0.000**
	Ártabro Gulf	0.004	0.006	–	0.013	0.000**	0.000**	0.894	0.000**	0.000**	0.000**	0.000**
	Cádiz	0.004	0.011	0.009	–	0.000**	0.000**	0.314	0.000**	0.000**	0.000**	0.000**
	Tenerife	0.053	0.064	0.063	0.045	–	0.291	0.000**	0.000**	0.000**	0.000**	0.000**
	Lanzarote	0.053	0.066	0.063	0.041	0.003	–	0.000**	0.000**	0.000**	0.000**	0.000**
Mediterranean Sea	Granada	0.009	0.001	– 0.005	0.004	0.038	0.034	–	0.000**	0.000**	0.000**	0.000**
	Ebro Delta	0.138	0.178	0.166	0.126	0.100	0.089	0.147	–	0.008	0.260	0.121
	Collioure	0.109	0.142	0.132	0.097	0.067	0.067	0.119	0.023	–	0.008	0.137
	Marseille	0.141	0.185	0.167	0.120	0.102	0.091	0.157	0.012	0.015	–	0.466
	Livorno	0.110	0.155	0.136	0.097	0.069	0.061	0.119	0.018	0.008	0.005	–

*Significance after FDR correction (*P* < 0.0033). **Significance after Bonferroni correction (*P* < 0.00091).

In order to increase the reliability of the study, the original microsatellite dataset (excluding locus Pe 11) was pared down to check the impact of the putative cryptic species on the genetic structure results of *P. elegans sensu stricto*. In fact, individuals belonging to the putative cryptic species presented a considerable amount of missing data in some loci, especially in loci Pe08 and Pe17. Thus, another STRUCTURE run was carried out removing these 69 individuals and the 4 individuals that could not be assigned to any cluster, so the microsatellite dataset exclusively contained genotypes of individuals considered as *P. elegans sensu stricto* (327 individuals). Delta*K* statistic distribution peaked at *K* = 2 (Supplementary Figure S1c), indicating again the partition of *P. elegans sensu stricto* samples into two clusters (Supplementary Figure S3a): one cluster joined Granada locality to the Atlantic localities (Ré Island, Santoña, Ártabro Gulf, Cádiz, Tenerife and Lanzarote), meanwhile the other cluster was formed by the Mediterranean localities (Almería, Ebro Delta, Collioure, Marseille, Livorno and Mallorca). The pattern of divergence between the sampling sites located on the Atlantic coast and those on the Mediterranean Sea was detected again for *P. elegans sensu stricto*. AMOVA was performed to test this two-group structure in this reduced dataset. When excluding the individuals of the putative cryptic species, differentiation among groups decreased (*FCT* = 0.094), explaining the 9.37% of the total variance (Supplementary Table S4).

By partitioning samples of *P. elegans sensu stricto* into two groups (Fig. 1b and Supplementary Figure S3a) according to the Bayesian clustering analysis, most of the individuals from Tenerife and Lanzarote were assigned to the Atlantic Ocean cluster as expected, but a considerable number of Canarian individuals could not be assigned to either of the two clusters (probability of membership to a cluster < 0.75). Assessing the results of the STRUCTURE analysis using the *P. elegans sensu stricto* dataset, a peak of *ΔK* and a plateau in the plot of *K* versus ln Pr(X|*K*) (Supplementary Figure S1d) were observed at *K* = 3, so the partition in three groups was plotted (Supplementary Figure S3b). With this grouping, the Mediterranean Sea cluster remained mainly invariable, whereas the Atlantic Ocean cluster was split into two subgroups, being then possible to clearly assign individuals from the Canary Islands localities to one of these two Atlantic clusters. Three-group AMOVA using this dataset unveiled that the 8.80% of the global variance is due to differences among groups, so the *FCT* value (*FCT* = 0.088) was lesser respecting to two-group AMOVA (Supplementary Table S4). Overall, the most likely genetic structure of *P. elegans sensu stricto* is two populations, one of them including the Atlantic localities and Granada, and the other encompassing the Mediterranean localities. Nevertheless, it is noteworthy to highlight that there is a substructure within the Atlantic population, with a subpopulation not exclusively but mainly formed by individuals from the Canary Islands. This substructure is in line with the genetic differentiation between Tenerife and Lanzarote and the remaining Atlantic localities suggested by the *FST* pairwise values. Finally, isolation-by-distance (IBD) was retrieved in *P. elegans sensu stricto* by the Mantel test, as genetic and geographical distances resulted to be significantly correlated (R = 0.37, *P* = 0.001). Another two Mantel tests were performed to test IBD within each population of *P. elegans sensu stricto*. No IBD was detected within the Mediterranean basin (R = 0.00, *P* = 0.750), meanwhile a significant relationship between genetic and geographical distances was found along the Atlantic population (R = 0.40, *P* = 0.015).

## Discussion

In this study, microsatellite markers were used for the analysis of the genetic diversity and population structure of the shrimp *P. elegans*. Levels of polymorphism were in line with those found in the counterpart common littoral shrimp *P. serratus*^25,29^. Sampling sites showed similar levels of genetic diversity (*Na*, *A*, *AR*, *Ho* or *He*). Thirty-one locality-locus combinations significantly deviated from HWE, mainly caused by heterozygote deficiency (29 out of the 31 cases) reflected in positive *FIS* values. Stutter-related scoring errors were discarded as the source of HWE departures because markers with ambiguous interpretation patterns were not included in the microsatellite set during the characterization work^27^ nor reported during the genotyping step here. Different causes for HWE deviations are frequently invoked such as inbreeding, Wahlund effect or null alleles. Inbreeding should equally affect all loci, generating similar heterozygote deficit across them. Despite global positive *FIS* values were obtained in all localities, great differences in *FIS* values across loci were observed, discarding inbreeding. Heterozygote deficit caused by subpopulation structure (Wahlund effect) could not be completely ruled out in localities where an arbitrary admixture of individuals from putative different species was found. Nevertheless, the presence of null alleles is the most likely explanation for the observed departures from HWE. The instability of the flanking sequences of microsatellites leads to some alleles could not be amplified and consequently dropped out, resulting in a homozygote excess. Although microsatellite null alleles are widespread, marine invertebrates have demonstrated particularly higher frequencies than other groups^30,31^. The 12 loci that deviated from HWE due to heterozygote deficit showed evidence of occurrence of null alleles, even though mainly in a low proportion. Only one locus, the locus Pe11, showed certain consistency in high null allele frequency estimates across localities and overall. For this reason, we decided to exclude locus Pe11 in population structure analyses as it was advised in González-Castellano et al*.*^27^.

Microsatellite data provided evidence for the existence of two clearly genetically distinct groups within *P. elegans.* Some individuals from Mediterranean localities showed a marked genetic divergence from the remaining specimens without following any geographical or oceanographic pattern. Since all shrimps from Granada, Almería, Collioure, Livorno and Mallorca were captured simultaneously in the same rockpools and unambiguously morphologically identified as *P. elegans* afterwards, we concluded that the explanation for the divergent individuals is the presence of a cryptic and sympatric species existing within *P. elegans*. Previous studies using mtDNA markers reported that some individuals from the Mediterranean Sea exhibited divergent haplotypes respecting the rest of the sampled collection, being those named *P. elegans* type III and considered as a cryptic species^8,12,13^. In fact, the individuals assigned with our microsatellite set to a cryptic species were confirmed to correspond with those *P. elegans* type III by their mtDNA COI sequences. The alternative hypothesis to explain this divergence in nuclear markers among our samples would be strong genetic structuring among individuals who live sympatrically and without exhibiting evident intrinsic reproductive barriers, what seems extremely unlikely.

Cryptic species are distributed among taxa, but they are particularly prone in the marine environment^32^, being detected within various species using microsatellite markers^33–35^. Here, the 400 sampled shrimps were assigned to one of the two unveiled genetic groups, i.e. *P. elegans sensu stricto* or cryptic species. Only two individuals from Granada and two from Mallorca could not be assigned to one specific cluster, most likely because they presented missing data at several loci. Consequently, no hybrids between *P. elegans sensu stricto* and the cryptic species were found. Hence, unknown reproductive isolating mechanisms between these two species should be acting to explain the absence of genetic hybrids. The main question to be solved is the origin of the cryptic species. Given that it was confined in the Mediterranean Sea, it should be originated there. The Mediterranean basin has a very complex geological history that largely contributed to its high level of biodiversity and endemism^15^. Reuschel et al*.*^8^ postulated that *P. elegans* type III specimens might have their origin during the Messinian Salinity Crisis in late Miocene (around 5.6 Myr ago), when the Strait of Gibraltar was closed, isolating the Mediterranean Sea from the Atlantic Ocean and determining the extensive desiccation of the Mediterranean basin^36^. According to Reuschel et al*.*^8^, isolated *P. elegans* type III ancestors had to survive in local refugia in the Mediterranean Sea and genetically diverge from the Atlantic population of *P. elegans,* giving rise to the cryptic species detected within *P. elegans* nowadays. With the re-opening of the Strait of Gibraltar, the Mediterranean basin was flooded again with Atlantic water, so Atlantic *P. elegans* individuals should be re-introduced, but they should no longer be able to reproduce with the cryptic species ancestors, since none hybrids are currently detected. Similarly, the Messinian Salinity Crisis was considered the origin of two sister crab species of the genus *Carcinus*^37^ and of two sister brittle star species of the genus *Ophiothrix*^38^. Our analyses using microsatellite loci support the existence of two species within *P. elegans*, providing nuclear evidence for the divergence signatures previously detected at mitochondrial level. Future efforts should be directed to clarify the origin of the cryptic species by phylogenetic or phylogenomic studies with precise calibration and to revise the taxonomy of *P. elegans* complex.

Regarding the population biology of *P. elegans sensu stricto* (*P. elegans* hereafter), a genetic structure was revealed in the whole study. Genetic differentiation analyses (Bayesian analysis, AMOVA and *FST* statistics) showed a pattern of two main populations: one composed by the Atlantic localities and Granada, and another composed by the Mediterranean localities. In other words, a clear geographic distinction between Atlantic and Mediterranean sampling sites was detected, with an extension of the Atlantic genotypes into Mediterranean Sea waters. Granada locality was genetically closer to the Atlantic localities than to the other Mediterranean localities. Therefore, a biogeographical barrier restricting the gene flow between these two populations should be located between Granada (Atlantic population) and Almería (Mediterranean population). Precisely, this genetic break matched with the location of the Almería-Orán Front, a semi-permanent dynamic oceanographic front consisted in two anticyclonic gyres that entails abrupt changes of temperature and salinity^39^. The Almería-Orán Front has been considered as the most important hydrographic boundary to gene flow between the Atlantic Ocean and Mediterranean Sea surface waters^11^. Indeed, it was confirmed that the Almería-Orán Front acts on the population structure of several species as in *P. elegans,* e.g. the cuttlefish *Sepia officinalis*^40^, some fish species^41^, the barnacle *Chthamalus montagui*^42^ or the wedge clam *Donax trunculus*^43^ among others (see review in Patarnello et al*.*^11^). Notwithstanding, it is known that population connectivity across the Atlantic–Mediterranean transition is reduced in other species by different oceanographic discontinuities, namely the Strait of Gibraltar^24,44^, the Balearic Front and the Ibiza Channel^45,46^. Most recently, in the congeneric common littoral shrimp *Palaemon serratus,* both mtDNA^47^ and nuclear^25^ markers have reported strong genetic differentiation among Atlantic and Mediterranean localities with an unusual phylogeographical break located west of the Strait of Gibraltar in the Gulf of Cádiz. In the present study, microsatellite data unravelled that the Almería-Orán Front was the only oceanographic discontinuity affecting the population structure of *P. elegans* across the Atlantic–Mediterranean transition*,* so our results agreed with the phylogeographic pattern described in Reuschel et al*.*^8^ using mitochondrial data. However, it is important to note that the Almería-Orán Front experiences seasonal fluctuations in intensity, size and position^39^ that might mitigate its effects as oceanographic barrier between the populations of *P. elegans*.

Differentiation was more complex within each group. Within the Mediterranean population, all localities showed no genetic differentiation among them in the light of our data. Our shrimp samples only covered the northern of the Western Mediterranean basin, so it would be interesting to analyse more Mediterranean localities to study population connectivity throughout the entire Mediterranean Sea. As for the Atlantic group, genetic analyses indicated some substructure within this population. Specifically, Canary Islands localities (Tenerife and Lanzarote) showed low but significant genetic differentiation from the remaining Atlantic localities (Ré Island, Santoña, Ártabro Gulf, Cádiz and Granada) as suggested by the pairwise *FST* values and also observed in the Bayesian analysis. Thus, the above results allowed us to report two subgroups within the Atlantic population: one formed by the South of France and the Iberian Peninsula localities; and another group comprising the Canary Islands localities. The lack of connectivity between these subgroups is consisted with the isolation-by-distance pattern observed along the Atlantic population of *P. elegans*. In this regard, lack of gene flow between Macaronesian Islands (Azores, Madeira, Canary Islands and Cape Verde) and continental locations has been also reported for various marine invertebrates, such as limpets of the genus *Patella*^48^, starfishes of the genus *Marthasterias spp*^49,50^*.* or the sea cucumber *Holothuria (Holothuria) mammata*^51^, as well as for fishes as the triplefin *Tripterygion delaisi*^52^ and the white seabream *Diplodus sargus*^53^. The remoteness of the Canary Islands from the Atlantic mainland sampling sites may explain the weak differentiation of Canarian *P. elegans* samples. The duration of the larval stage compared to the great distance that separates the Canary Islands and the continent may not be enough to prevent this differentiation. Related to this, present-day surface currents in Macaronesia, mainly the Azores Current and the Canary Current^54^, could also favour the relative isolation of the Canary Islands by disrupting larval dispersion. A more extensive sampling would be needed to reveal whether there is more genetic structuring along the Atlantic distribution of this shrimp.

Finally, the microsatellite loci used in this study were isolated and characterized from Atlantic individuals^27^, so they are specific markers for *P. elegans sensu stricto*. In order to study the genetic structure of the cryptic species found in the Mediterranean Sea, firstly we recommend revising its status as an independent new species, and then to develop novel specific microsatellite markers to carry out population analyses with them. De novo development of species-specific microsatellites is strongly recommended. Cross-amplification from congeneric species is not generally feasible because inherent problems like allele size homoplasy, polymorphisms biases, null alleles presence, broken repeats motifs or amplification of non-orthologous loci could arise^55–58^.

In conclusion, this study encompasses the first assessment of the genetic diversity and population structure of the shrimp *P. elegans* using microsatellite markers. Two clearly genetically distinct groups were detected within what is taxonomically considered as *P. elegans,* indicating the existence of a Mediterranean cryptic species within *P. elegans.* Furthermore, *P. elegans sensu stricto* is genetically structured in two populations: the Atlantic population and the Mediterranean population. The genetic break that separates these populations was found across the Atlantic–Mediterranean transition, matching with the location of the Almería-Orán Front. The microsatellite loci were sensitive enough to also reveal certain differentiation within the Atlantic population mainly involving individuals from the Canary Islands, which may be explained by an isolation-by-distance pattern.

## Material and methods

**Figure 2:**
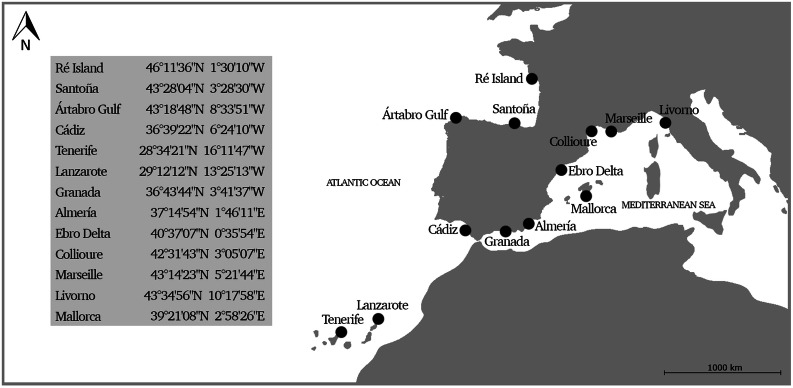
Geographical location and coordinates of the sampling sites of *Palaemon elegans*. Map was newly generated by the authors using AutoCAD software.

Specimens of *P. elegans* used in this study were collected inshore between 2012 and 2019 from 13 European sampling sites: Ré Island (France), Santoña (Spain), Ártabro Gulf (Spain), Cádiz (Spain), Tenerife (Spain), Lanzarote (Spain), Granada (Spain), Almería (Spain), Ebro Delta (Spain), Collioure (France), Marseille (France), Livorno (Italy) and Mallorca (Spain) (Fig. 2). Animals were captured in intertidal rockpools with a fish trap and ethanol-stored at laboratory. Morphological identification of the 400 sampled shrimps was thoroughly carried out according to González-Ortegón & Cuesta^1^. Genomic DNA was extracted from 25 mg of abdominal muscle tissue using the NZY Tissue gDNA Isolation kit (NZYTech) following the manufacturer’s instructions. Extracted DNA quality and concentration were determined with the NanoDrop ND-1000 spectrophotometer (Thermo Fisher Scientific).

Individual genotypes were obtained for 21 polymorphic microsatellite loci (Pe01-Pe21: MH078079-MH078099) previously developed by González-Castellano et al*.*^27^ for *P. elegans* and following the wet-lab protocol described there. Fluorescently labelled polymerase chain reaction (PCR) products were run on a 3130xl Genetic Analysis System (Applied Biosystems) for fragment analysis in the Scientific Research Support Services (University of A Coruña), using GeneScan 500 (− 250) ROX internal size standard (Applied Biosystems). Geneious 8.0.5^59^ was used for fluorescent profiles analysing and allele peaks calling.

Genetic variability was assessed with standard descriptive statistics. Number of alleles (*Na*), mean number of alleles (*A*), percentage of polymorphism (*P*), observed heterozygosity (*Ho*), expected heterozygosity (*He*) and deviations from Hardy–Weinberg equilibrium (*HWE*) for each locus and/or locality were estimated using GenAlEx 6.5^60^. Significance levels were adjusted applying the sequential Bonferroni correction whenever multiple tests were performed. Allelic richness per locality was calculated using FSTAT 2.9.4^61^. GENEPOP 4.2^62^ was used to estimate inbreeding coefficient (*FIS*) and to test linkage disequilibrium between pairs of loci using Fisher's exact test. Significance was computed by a Markov chain method using 10,000 dememorizations, 5,000 batches and 5,000 iterations per batch. Departures from selective neutrality were evaluated for each locus applying the Ewens-Watterson test using the algorithm given in Manly^63^ and implemented in POPGENE 1.32^64^ after 1,000 simulations. Null allele frequency was estimated using FreeNA^28^ and the EM algorithm^65^ with a number of bootstrap replicates fixed to 10,000.

Genetic structure was assessed using the Bayesian model-based clustering method implemented in STRUCTURE 2.3.4^66^. The admixture model with correlated allele frequencies between populations^67^ was used. A burn-in of 100,000 iterations followed by 300,000 iterations for parameter estimation was set up. Each simulation was run 10 times, exploring values for *K* ranging from 1 to *N* + 2, where *N* was the number of localities. Results were processed using STRUCTURE HARVESTER^68^. The best partition of the data was determined by examining both the log probability of the data (ln Pr(*X*|*K*)) and the Δ*K* statistic following Evanno, Regnaut, & Goudet^69^. CLUMPP 1.1.2^70^ was used to permute the admixture coefficients for the several independent runs resulting for the chosen *K*-value. STRUCTURE PLOT 2.0^71^ was employed to visualize the output from CLUMPP. Genetic discrimination among samples was additionally assessed through a factorial correspondence analysis (FCA) using GENETIX 4.05^72^.

Genetic differentiation was estimated as pairwise *FST* values (10,000 permutations) between localities using ARLEQUIN 3.5.1.2^73^. Significance level was adjusted applying sequential Bonferroni correction for multiple comparisons and false discovery rate (FDR) correction (B-Y method as described in Narum^74^). Several hierarchical locus-by-locus analyses of molecular variance (AMOVA) were carried out using ARLEQUIN 3.5.1.2^73^ in order to estimate partitioning of genetic variance among groups (*FCT*), among localities within groups (*FSC*) and within localities (*FST*). Individuals were pooled according to a geographic/oceanographic criterion or based on the results previously obtained with STRUCTURE to test different groupings.

Lastly, the relationship between genetic distance and geographic distance was assessed with a Mantel test^75^ to test isolation-by-distance (IBD) using the program ISOLDE implemented in GENEPOP 4.2^62^ with 10,000 permutations. Pairwise genetic differentiation among localities was estimated using linearized *FST* [*FST*/(1 − *FST*)] to construct a genetic distance matrix. Pairwise geographic distance between localities was measured in kilometres following the coastline in Google Earth (Google Inc.) to create a geographic distance matrix.

## Supplementary information

Supplementary file1 (DOCX 18 kb)

Supplementary file2 (XLSX 36 kb)

Supplementary file3 (PDF 380 kb)

Supplementary file4 (TXT 39 kb)

## Data Availability

Accession codes: Sequences containing the microsatellite loci were deposited in GenBank under Accession Numbers MH078079-MH078099. Genotype dataset generated during the current study is available from the corresponding authors on reasonable request.
