# Supplementary material for: Genetic diversity and population structure of the rockpool shrimp Palaemon elegans based on microsatellites: evidence for a cryptic species and differentiation across the Atlantic–Mediterranean transition
Source: Sci Rep. 2020 Jul 1;10:10784. doi: 10.1038/s41598-020-67824-7 (PMC7329806; doi:10.1038/s41598-020-67824-7)
Supplement: Supplementary file 1 — Supplementary file1 (DOCX 18 kb) [file 41598_2020_67824_MOESM1_ESM.docx]

Genetic diversity and population structure of the rockpool shrimp *Palaemon elegans* based on microsatellites: evidence for a cryptic species and differentiation across the Atlantic-Mediterranean transition

Inés González-Castellano*, Jorge González-López, Ana M. González-Tizón, Andrés Martínez-Lage*

Universidade da Coruña, Departamento de Biología and Centro de Investigaciones Científicas Avanzadas (CICA), 15071 A Coruña, Spain

*Corresponding authors: [ines.gonzalez.castellano@udc.es](mailto:ines.gonzalez.castellano@udc.es) Tel.: +34881015594; [andres@udc.es](mailto:andres@udc.es) Tel.:+34 881012020

**Supplementary Material:**

**Supplementary Figure S1. Methods to evaluate the most likely partition of the data in the STRUCTURE analysis.** Analysis including the 400 sampled individuals: (a) Evanno's ad hoc statistic, deltaK as a function of K, over ten replicates and (b) mean log probability of data Pr(X/K), bars represent standard deviation. Analysis including only individuals considered as *Palaemon elegans* sensu stricto (327 individuals): (c) Evanno's ad hoc statistic, deltaK as a function of K, over ten replicates and (d) mean log probability of data Pr(X/K), bars represent standard deviation.

**Supplementary Figure S2. Factorial correspondence analysis (FCA) based on 20 microsatellite loci.** Analysis including the 400 sampled individuals and computed using GENETIX. Each color depicts a locality and each triangle represents an individual.

**Supplementary Figure S3. Bayesian assignment probabilities for (a) *K* = 2 and (b) *K* = 3 revealed by STRUCTURE based on 20 microsatellite loci and only including individuals considered as *P. elegans* sensu stricto (327 individuals).** Each color depicts a cluster and each vertical bar represents an individual with the probability of membership to a cluster.

**Supplementary Table S1. Characterization of the 21 microsatellite loci for *Palaemon elegans*. 5′ tails attached to reverse primers are in brackets.**

Notes. *N*, number of genotyped individuals; *Na*, number of alleles per locus, with private alleles in brackets; *Ho*, mean observed heterozygosity per locus; *He*, mean expected heterozygosity per locus; Null, null allele frequency.

**Supplementary Table S2. Parameters of genetic diversity in *Palaemon elegans* per sampling site and per microsatellite locus.**

Notes. *N*, Number of genotyped individuals; *Na*, number of alleles, with private alleles in brackets; *Ho*, observed heterozygosity; *He*, expected heterozygosity; Null, null allele frequency; *FIS*, inbreeding coefficient. Significant departure from Hardy–Weinberg equilibrium (HWE) after the sequential Bonferroni correction is indicated next to *Ho* value as asterisk (*P <* 0.000037).

**Supplementary Table S3. Pairwise *FST* values (below the diagonal) revealed by the microsatellite dataset (20 loci), and the corresponding *P*- values (above the diagonal). Sampling sites were subdivided grouping *Palaemon elegans* sensu stricto and putative cryptic species individuals separately.**

Notes. *Significance after FDR correction (*P* < 0.0031). **Significance after Bonferroni correction (*P* < 0.00071).

**Supplementary Table S4. Analysis of molecular variance (AMOVA) based on different grouping hypotheses and only including individuals considered as *P. elegans* sensu stricto (327 individuals).**

Notes. (a) No grouping indicates that localities were analysed separately. (b) For two-group analysis, localities were pooled as Ré Island-Santoña-Ártabro Gulf-Cádiz-Tenerife-Lanzarote-Granada (Atlantic Ocean) and Almería-Ebro Delta-Collioure-Marseille-Livorno-Mallorca (Mediterranean Sea). (c) For three-group analysis, Ré Island-Santoña-Ártabro Gulf-Cádiz-Granada (Atlantic Ocean), Almería-Ebro Delta-Collioure-Marseille-Livorno-Mallorca (Mediterranean Sea) and Tenerife-Lanzarote (Canary Islands) each formed a group.

**Supplementary File S1. Alignment of COI nucleotide sequences from individuals belonging to the putative cryptic species.** Notes. 64 individuals belonging to the putative cryptic species were included and sampling site from which each individual was collected is indicated as abbreviation in the sequence name as: GRA, Granada; ALM, Almería; COL, Collioure, LIV, Livorno; and MALL, Mallorca.
