# Supplementary material for: Genetic diversity and population structure of the rockpool shrimp Palaemon elegans based on microsatellites: evidence for a cryptic species and differentiation across the Atlantic–Mediterranean transition
Source: Sci Rep. 2020 Jul 1;10:10784. doi: 10.1038/s41598-020-67824-7 (PMC7329806; doi:10.1038/s41598-020-67824-7)
Supplement: Supplementary file 3 — Supplementary file3 (PDF 380 kb) [file 41598_2020_67824_MOESM3_ESM.pdf]

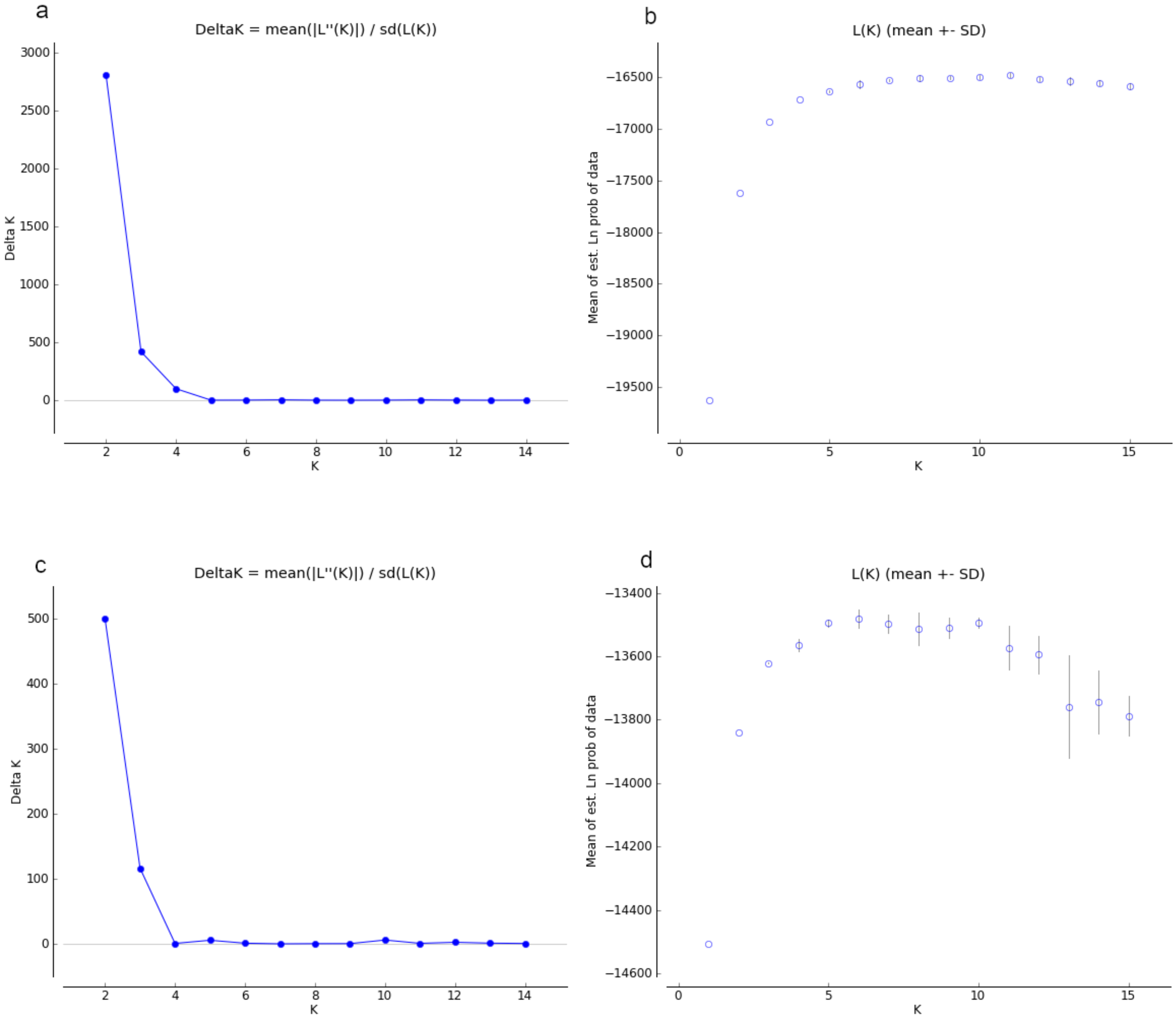

Supplementary Figure S1. Methods to evaluate the most likely partition of the data in the STRUCTURE analysis. Analysis including the 400 sampled individuals: (a) Evanno's ad hoc statistic, deltaK as a function of K, over ten replicates and (b) mean log probability of data Pr(X/K), bars represent standard deviation. Analysis including only individuals considered as *Palaemon elegans* sensu stricto (327 individuals): (c) Evanno's ad hoc statistic, deltaK as a function of K, over ten replicates and (d) mean log probability of data Pr(X/K), bars represent standard deviation.

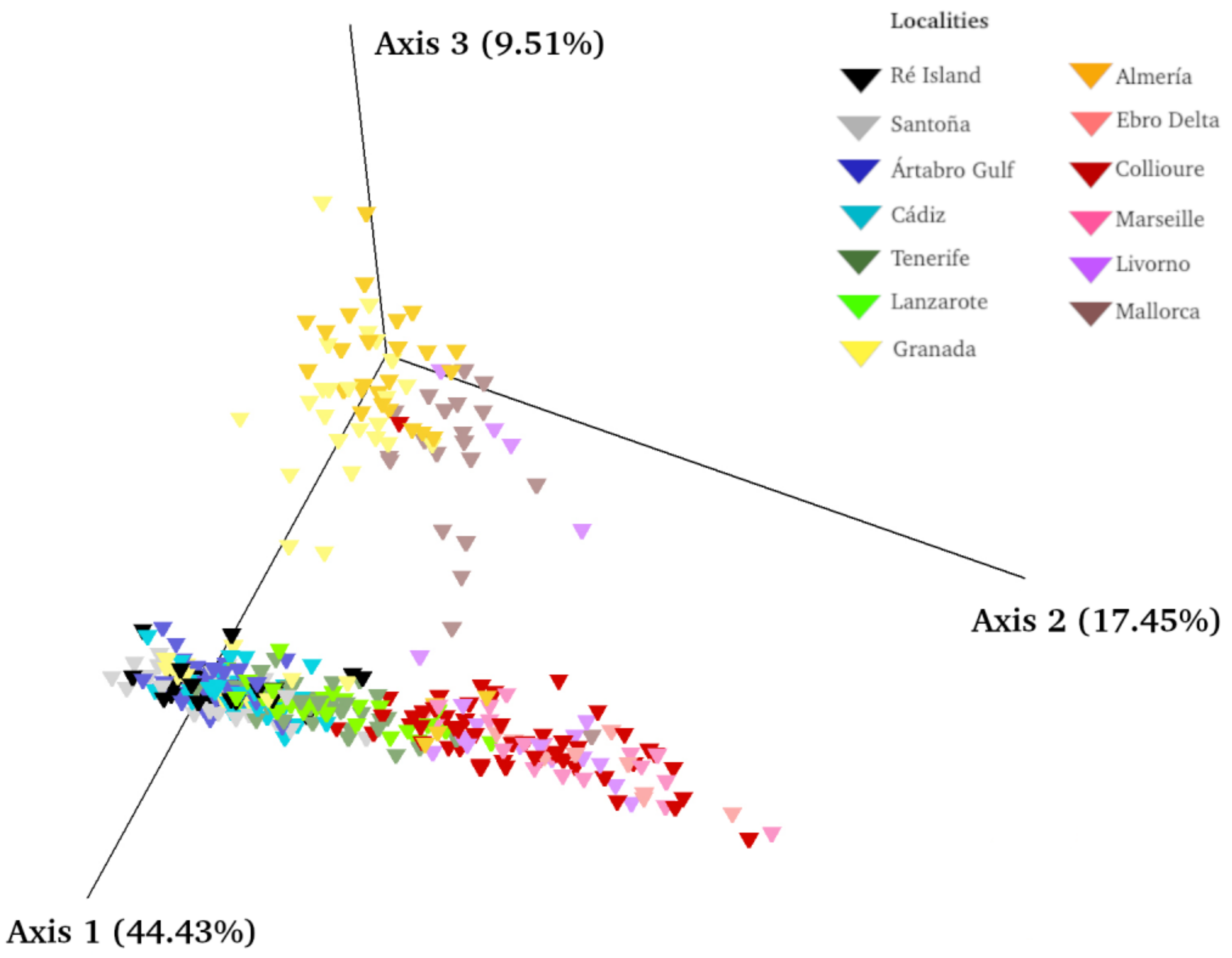

Supplementary Figure S2. Factorial correspondence analysis (FCA) based on 20 microsatellite loci. Analysis including the 400 sampled individuals and computed using GENETIX. Each color depicts a locality and each triangle represents an individual.

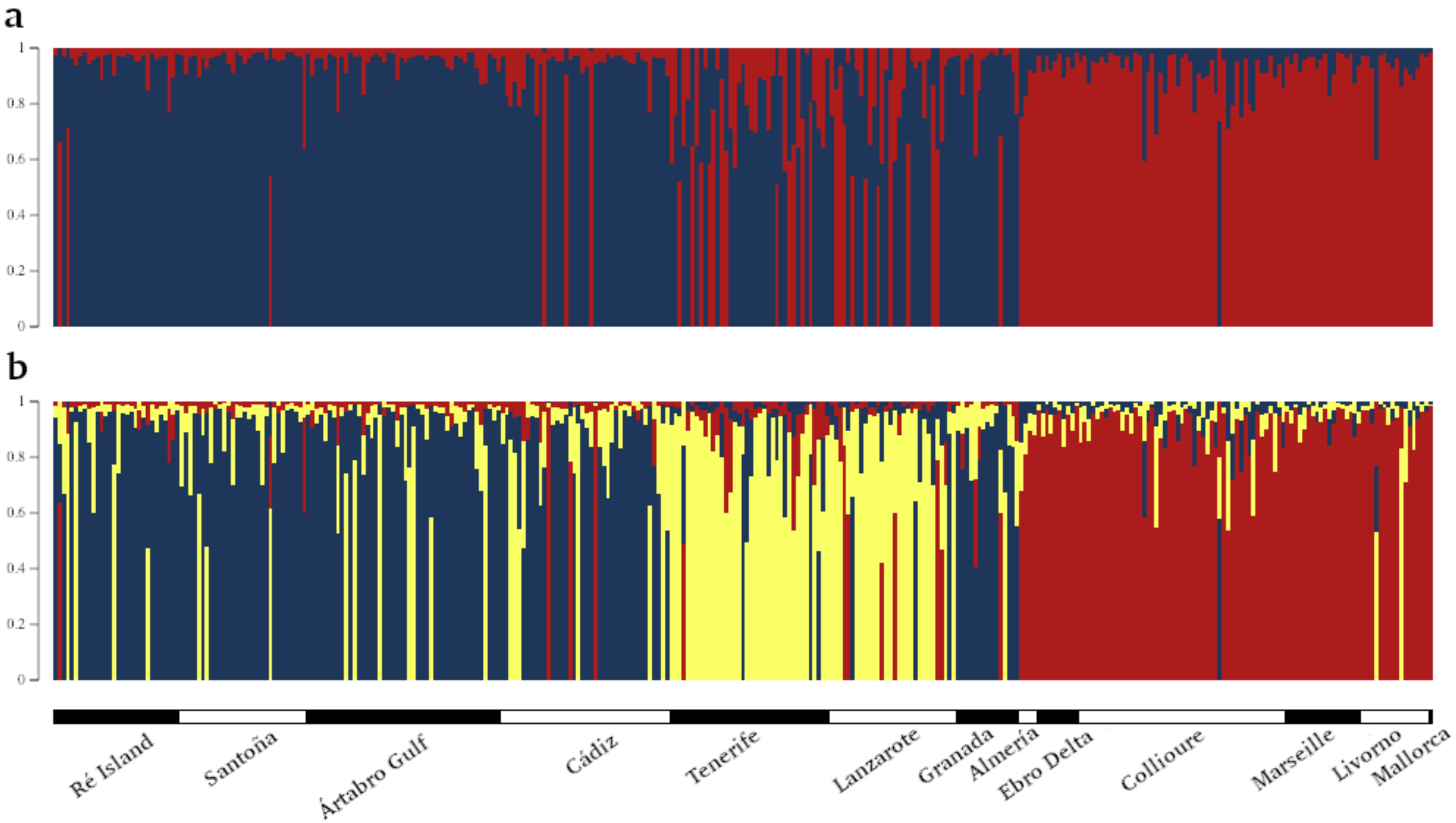

Supplementary Figure S3. Bayesian assignment probabilities for (a) K = 2 and (b) K = 3 revealed by STRUCTURE based on 20 microsatellite loci and only including individuals considered as *P. elegans* sensu stricto (327 individuals). Each color depicts a cluster and each vertical bar represents an individual with the probability of membership to a cluster.
